# Adding Blue to Red Supplemental Light Increases Biomass and Yield of Greenhouse-Grown Tomatoes, but Only to an Optimum

**DOI:** 10.3389/fpls.2018.02002

**Published:** 2019-01-14

**Authors:** Elias Kaiser, Theoharis Ouzounis, Habtamu Giday, Rachel Schipper, Ep Heuvelink, Leo F. M. Marcelis

**Affiliations:** Horticulture and Product Physiology Group, Wageningen University & Research, Wageningen, Netherlands

**Keywords:** LED, biomass, blue light, red light, photosynthesis, tomato, greenhouse, yield

## Abstract

Greenhouse crop production in northern countries often relies heavily on supplemental lighting for year-round yield and product quality. Among the different spectra used in supplemental lighting, red is often considered the most efficient, but plants do not develop normally when grown solely under monochromatic red light (“red light syndrome”). Addition of blue light has been shown to aid normal development, and typical lighting spectra in greenhouse production include a mixture of red and blue light. However, it is unclear whether sunlight, as part of the light available to plants in the greenhouse, may be sufficient as a source of blue light. In a greenhouse high-wire tomato (*Solanum lycopersicum*), we varied the percentage of blue supplemental light (in a red background) as 0, 6, 12, and 24%, while keeping total photosynthetically active radiation constant. Light was supplied as a mixture of overhead (99 μmol m^-2^ s^-1^) and intracanopy (48 μmol m^-2^ s^-1^) LEDs, together with sunlight. Averaged over the whole experiment (111 days), sunlight comprised 58% of total light incident onto the crop. Total biomass, yield and number of fruits increased with the addition of blue light to an optimum, suggesting that both low (0%) and high (24%) blue light intensities were suboptimal for growth. Stem and internode lengths, as well as leaf area, decreased with increases in blue light percentage. While photosynthetic capacity increased linearly with increases in blue light percentage, photosynthesis in the low blue light treatment (0%) was not low enough to suggest the occurrence of the red light syndrome. Decreased biomass at low (0%) blue light was likely caused by decreased photosynthetic light use efficiency. Conversely, decreased biomass at high (24%) blue light was likely caused by reductions in canopy light interception. We conclude that while it is not strictly necessary to add blue light to greenhouse supplemental red light to obtain a functional crop, adding some (6–12%) blue light is advantageous for growth and yield while adding 24% blue light is suboptimal for growth.

## Introduction

In northern countries, low light intensities and short days persist for large parts of the year. In greenhouse production, supplemental lighting is often used to maintain year-round production and product quality (Davis and Burns, 2016). High-pressure sodium (HPS) lamps are currently the predominant greenhouse lighting source. However, HPS are neither spectrally (deficient in blue) nor energetically optimal (Gomez et al., 2013), and light-emitting diodes (LEDs) are emerging as a promising alternative (Mitchell et al., 2015; Davis and Burns, 2016). LEDs are solid-state semi-conductor devices emitting narrow-bandwidth light, with high life expectancy and low heat radiation. These features enable an optimization of light spectrum for plant growth and development, lower energy costs and a placement of lamp fixtures closer to the crop (Bourget, 2008; Davis and Burns, 2016).

Adding artificial light on top of the canopy (overhead lighting) is common for HPS and LED installations alike. However, light intensity decreases exponentially within a crop canopy, resulting in strong light intensity gradients between the top and bottom of the crop (Gomez et al., 2013), and possibly suboptimal light distribution for optimal whole-canopy carbon gain. Partial replacement of overhead by intracanopy lighting has potential for improving light distribution in the canopy. For intracanopy lighting, lamps illuminate plants from the side rather than from the top at lower parts of the canopy (Nelson and Bugbee, 2014; Davis and Burns, 2016). Growth under a combination of overhead and intracanopy lighting has been found to be higher (Frantz et al., 2000; Hovi-Pekkanen and Tahvonen, 2008) or similar (Trouwborst et al., 2010; Dueck et al., 2012; Gomez et al., 2013; Gomez and Mitchell, 2016) to growth under overhead lighting alone. Differences between studies may partially be explained by different crop architecture (e.g., profiles of leaf density and leaf angle).

Red light (600–700 nm) is the most efficient color for powering photosynthesis, while the energy content of red photons is relatively low (McCree, 1972; Paradiso et al., 2011; Hogewoning et al., 2012), making red the preferred color for supplementary lighting. However, growth and development of plants grown strictly under monochromatic red light are seriously hampered (“red light syndrome”), with symptoms including leaf curling and decreases in photosynthetic capacity, leaf thickness and leaf pigmentation (Hogewoning et al., 2010b; Ouzounis et al., 2016; Trouwborst et al., 2016; Zhang et al., 2018). Adding blue light (400–500 nm) has been shown to suppress these symptoms (Hogewoning et al., 2010b; Ouzounis et al., 2016; Trouwborst et al., 2016). Therefore, blue light is usually added in plant commercial greenhouse lighting. However, this is solely based on experiments in climate chambers without a background of solar light. It has not been quantified if and how much blue light is minimally required to suppress the red light syndrome in greenhouse cultivation.

Additionally, leaf photosynthetic capacity has been shown to increase with the percentage of blue light (up to 50% blue light in a red light background; Hogewoning et al., 2010b). Thus, blue light produces “sun-type” leaves even when overall light intensity is low. Relatively higher photosynthesis rates at high light intensities might in turn increase growth and yield, and ameliorate the costs of adding blue light to supplemental lighting. On the other hand, in greenhouses, natural sunlight supplies part of the light available to crops, and this includes 27–31% of blue light (Bird and Riordan, 1986; Hogewoning et al., 2010a), which may be sufficient even in winter. This leads to three questions: (i) Is blue supplemental lighting in greenhouses necessary? (ii) If so, how much blue supplemental lighting is necessary to suppress the red light syndrome? (iii) How much blue supplemental lighting is necessary for optimal yield?

The role of supplemental blue light in greenhouses with a background of solar light has so far not been investigated. The objectives of this study were to characterize the effects of blue in a red supplemental light and sunlight background on plant growth and development, under natural light intensities closely resembling an average Dutch winter. Further, we aimed to analyze the morphological and physiological processes through which the treatment effects could be explained.

## Materials and Methods

### Plant Material and Growth Conditions

Tomato (*Solanum lycopersicum* L. “Foundation”; Nunhems, Haelen, the Netherlands) seeds were sown on December 20, 2016 in potting soil (“Zaai/Stek Medium,” Horticoop, Slingerland Potgrond, Katwijk, Netherlands) and germinated at the glasshouse facilities of Wageningen University, Netherlands (52°N, 5.5°E). 14 days after germination, seedlings were transferred to 10 × 10 × 10 cm stonewool blocks (Grodan, Roermond, Netherlands) and grown in a glasshouse compartment with 22/16°C day/night temperature. Overhead supplemental lighting (175 μmol m^-2^ s^-1^ photosynthetically active radiation, PAR) was applied daily for 16 h using 600 W HPS lamps (Philips, Eindhoven, Netherlands). On February 10, 43 days after sowing, when plants were 34 cm tall, they were transferred to the experimental glasshouse compartment, and treatments were started.

Plant growth was managed close to common standards of Dutch growers. Plants were grown on stonewool slabs (Grodan) for 111 days in a double row “high wire” system at 2.4 plants m^-2^. The distance between the centers of the double rows was 150 cm. Climate set points were as follows: temperature 22/16°C (day/night), relative humidity 78%, CO_2_ partial pressure 500 μbar. Up to the sixth truss, all but six flower buds were removed. After truss no. six had been formed, a side stem was retained on each plant, doubling the stem density to 4.8 m^-2^. After anthesis of the second truss, leaves below the lowest ripe truss were removed from the canopy weekly. Once plants reached a threshold distance below overhead LED (38 cm), their stems were lowered weekly to keep their apices at a constant distance from the lamps. Overhead lamps were switched on 16 h before sunset, and switched off at sunset: throughout the experiment, switching on of lamps gradually changed from 1:40 to 6:40, and switching off changed from 17:40 to 22:40. Additionally, lamps were switched off when global radiation outside the greenhouse exceeded 450 W m^-2^, and switched on again when it fell below 250 W m^-2^. Intracanopy lamps were regulated identically to overhead lamps, except that they were not used during the first 5 days of the experiment, and that their use was gradually increased, by 1 h day^-1^, thereafter (i.e., 21 days after starting the experiment, photoperiod of all lamps was identical). All side walls of the greenhouse compartment were closed off using a reflective screen, to prevent light pollution from neighboring compartments. A standard nutrient solution for tomato was used (12.4 mM NO_3_^-^, 7.2 mM K^+^, 4.1 mM Ca^2+^, 3.3 mM SO_4_^2-^, 1.8 mM Mg^2+^, 1.2 mM NH_4_^+^, 1.1 mM PO_4_^3-^, 30 μM BO_3_^3-^, 25 μM Fe^3+^, 10 μM Mn^2+^, 5 μM Zn^2+^, 0.75 μM Cu^+^, and 0.5 μM MoO_4_^2-^; Yara Benelux B.V., Vlaardingen, Netherlands). Electrical conductivity (2.1 dS m^-1^) and pH (5.5) of the irrigation solution were monitored and adjusted daily.

### Treatments

**FIGURE 1:**
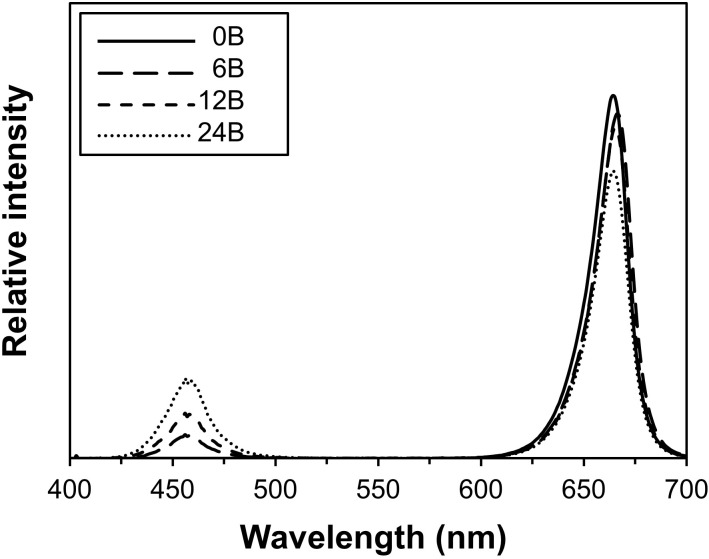
Combined spectral output of overhead and intracanopy lamps in the four blue/red treatment combinations.

Four combinations of blue and red supplemental light were obtained by combining several LED light sources, peaking at 445 and 665 nm, respectively (Figure 1). These combinations resulted in intended treatments of 0, 6, 12, and 24% of blue light in a red light background (referred to as 0B, 6B, 12B, and 24B, hereafter). Overhead supplemental lighting was provided by Greenpower PM-B150LO, Greenpower PM-DR150, Greenpower TL-DRBLBHO and Greenpower TL-DRBMBHO modules (Philips, Eindhoven, the Netherlands) and intracanopy lighting was provided by Greenpower PM-B150LO, Greenpower PM-B150MB, Greenpower PM-DR150 and Greenpower interlighting DR/B modules (Philips). The greenhouse compartment was split in two, a front and a rear half (as seen from the door), by the use of a white/black/white double plastic screen. In each half, a full repetition of the experiment was applied: in the front half, %B increased from left to right, in the rear half, %B increased from right to left. Plants were grown in rows from the front to the back of the compartment. Plots within each repetition were separated by a border row. For intracanopy lighting, two strings of Greenpower modules were positioned between double rows of plants. For the first 53 days of treatment (DOT), the distance between gutter and LED strings was 108 cm for the lower and 153 cm for the upper string. Thereafter, both strings were raised by 25 cm to account for plant elongation.

### Light Intensity Measurements

Vertical PAR distribution in the empty greenhouse was measured using a 1 m long line quantum sensor (LI-191SA, LI-COR Biosciences, Lincoln, NE, United States). At night, the sensor was positioned longitudinally in the middle of the path (75 cm from the center of each gutter), and PAR emitted by overhead and intracanopy LED lamps was measured separately, at regular intervals of 40–50 cm along a vertical plane. When measuring PAR from intracanopy lamps, the quantum sensor was turned toward those lamps (rotated 90° to the side) and overhead lamps were turned off, while the sensor was turned upward and intracanopy lights were turned off when measuring PAR from overhead lamps. These measurements were conducted in both 0B and 24B plots (four plots in total) and later averaged for daily light integral calculations (see below). The PAR spectrum emitted by LED lamps was measured in all plots. Global radiation (W m^-2^) above the greenhouse was measured continuously using a solarimeter (Kipp en Zonen, Delft, Netherlands). The fraction of PAR in the total global radiation was assumed to be 47% (Britton and Dodd, 1976), and the conversion factor from energy flux to quantum flux in the PAR region of sunlight was assumed to be 4.57 μmol J^-1^ (McCree, 1972). The transmissivity of the greenhouse was determined as 26%, by measuring PAR above the greenhouse, and just above the canopy, on a cloudy day. Greenhouse transmissivity was comparably low because red LED fixtures in the 0B treatments had a low output, necessitating the installation of many overhead fixtures. In the other treatments, wooden slats of the same dimensions were installed to ensure equal transmissivity in all treatments.

### Calculation of Daily Light Integral and Realized Spectrum

Realized blue light percentage was calculated daily by summing up daily light integrals (DLI, mol photons m^-2^ d^-1^) from overhead and intracanopy modules as well as from sunlight, and then calculating the percentage of blue as a fraction of DLI of each light source. The reference height for all calculations was the height of the fully grown canopy, and was 38 cm below the lamps (296 cm above the gutter). Light intensity at the reference height resulting from sunlight and overhead lighting was integrated daily (*DLI*_R_). Overhead LEDs supplied 123 μmol photons m^-2^ s^-1^ at reference height. As light intensity of overhead lamps and sunlight decreased exponentially within the empty greenhouse, light intensity at the top of the plant (P, distance between top of plant and gutter, in cm) was calculated as:

(1) $${DLI}_{P}={DLI}_{R}*{0.4238e}^{0.0029*P}$$

Parameters in Eq. 1 were determined from measurements of the light intensity profile in the empty greenhouse. Plants were assumed to elongate at a rate of 3.7 cm d^-1^ (based on plant length measurements after 0, 40, and 111 DOT), until hitting the reference height (on 73 DOT), after which plant height remained constant (by lowering plants’ apices every week). Intracanopy lighting resulted in a bell-shaped vertical light intensity profile with a maximum light intensity of 86 μmol m^-2^ s^-1^ (measured at 75 cm distance from lamps, without plants, with sensor facing the lamps). The DLI from intracanopy lighting was calculated daily as an average intensity along the height of the plant, multiplied by the height of the plant and divided by the width of the corridor between intracanopy modules on both sides of the plant (1.5 m), in order to express DLI from intracanopy lighting per m^2^ ground area. The DLI and %B received from intracanopy lighting was calculated daily, based on linear interpolation from measured values. Incident % blue light was calculated as:

(2) $$\%B=\frac{({DLI}_{o}*B_{o})+({DLI}_{i}*B_{i})+({DLI}_{s}*f_{dif}*B_{s_{dif}})+\left({DLI}_{s}*(1-f_{dif})*B_{s_{dir}}\right)}{\left({DLI}_{o}+{DLI}_{s}+{DLI}_{s}\right)}$$

Where, *DLI*_o_, *DLI*_i_ and *DLI*_s_ are the DLIs incident from overhead LEDs, intracanopy LEDs and sunlight, respectively. *B*_o_, *B*_i_, *B*_S_dif__ and *B*_S_dir__ are %B of overhead LEDs, intracanopy LEDs, diffuse sunlight (31.1%) and direct sunlight (27.9, as measured by Hogewoning et al., 2010a), respectively. The fraction of diffuse light in the sunlight spectrum (*f*_dif_) was calculated using daily data of direct and diffuse global radiation from the weather station “de Veenkampen” in Wageningen.

### Destructive Measurements

After 40 (intermediate harvest) and 111 DOT (final harvest), three complete plants per plot were destructively harvested. Stem length, number of leaves, and totals of leaf area, as well as leaf, stem and fruit dry weights (DW) were recorded on each occasion. Ripe fruits and old leaves were picked weekly from 57 DOT until final harvest, and their dry weights were recorded thereafter. Fruits were dried at 50°C for 24 h and then at 105°C for 48 h, while leaves were dried at 80°C for 24 h. Measurements were conducted on three plants per plot (six plants per treatment). Leaf area was determined with a leaf area meter (LI-3100; LI-COR).

### Gas Exchange and Chlorophyll Fluorescence

#### Light and CO_2_ Response Curves

After 25–27 DOT, the response of net photosynthesis rate (*A*; μmol m^-2^ s^-1^) and chlorophyll fluorescence to light intensity and leaf internal CO_2_ partial pressure (C_i_) was determined on leaf 5 (counting from above; leaf 1 was defined as ≥5 cm length). Measurements were performed on three plants per plot using the LI-6400 photosynthesis system (LI-COR), equipped with the 6400-40 fluorescence cuvette (enclosed leaf area: 2 cm^2^). Leaves were enclosed in the cuvette at 1500 μmol m^-2^ s^-1^ PAR, 2000 ± 2 μbar CO_2_ partial pressure, 23 ± 0.2°C cuvette temperature, 70 ± 5% RH and a flow rate of 400 μmol s^-1^. %B in the measuring cuvette was set to 0, 6, 12, and 20–24% in 0B, 6B, 12B, and 24B, respectively. After waiting for *A* to stabilize (∼15 min), CO_2_ partial pressure was decreased stepwise to 1500, 1000, 800, 600, 400, 300, 200, 100, and 50 μbar, while all other environmental variables were kept constant. Then, CO_2_ partial pressure and light intensity were raised to 400 μbar and 2000 μmol m^-2^ s^-1^ respectively, and after *A* had stabilized (∼10 min), light intensity was decreased stepwise to 1500, 1000, 800, 600, 400, 200, 150, 100, and 50 μmol m^-2^ s^-1^. At each CO_2_ or light intensity step (2–3 min duration), *A* was stabilized and values of CO_2_ and H_2_O measured by the infrared gas sensor of the sample cell were calibrated against those of the reference cell (“matching”). Then, *A* and C_i_ were logged for 30 s at intervals of 5 s, at an A/D signal averaging of 10 s. These values were averaged to increase accuracy. Additionally, operating (F_s_) and maximal (F_m_’) chlorophyll fluorescence yields were recorded at each light intensity step, using the multi-phase flash protocol (Loriaux et al., 2013). The intensity of the saturating flash was 10.000–14.000 μmol m^-2^ s^-1^, durations of the three phases were 0.4, 0.6, and 0.3 s respectively, and flash intensity decreased by 60% in phase 2. After the light response curve was finished, leaves were dark-adapted for 20 min. Then, minimum (F_o_) and maximum (F_m_) dark-adapted chlorophyll fluorescence yields were determined.

#### Diurnal Time Courses of Gas Exchange

After 32–33 DOT, instantaneous *A* and PAR were measured on leaf 5 using the LI-6400 photosynthesis system with a transparent leaf cuvette (6 cm^2^). Measurements were conducted between 8:00 and 18:30, and were repeated on the same leaf every 2 h throughout the day (6 time points). Cuvette temperature, CO_2_ partial pressure and relative humidity were the same as during measurements of light response curves. At each measurement, after CO_2_ and H_2_O partial pressures in the cuvette had equilibrated (1–2 min), gas exchange was logged every 5 s for 30 s; those values were averaged later.

#### Calculation of Photosynthesis Parameters

From light response curves, *A*_sat_ (light-saturated *A*), Φ_CO2_ (quantum yield) and Θ (curvature parameter), were determined from a non-rectangular hyperbola formula after Ögren and Evans (1993). Day respiration (R_d_; μmol m^-2^ s^-1^) was determined by calculating the intercept of the linear regression (*R*^2^ > 0.98 in all cases) between *A* and electron transport limiting light intensities (range: 50–150 μmol m^-2^ s^-1^). Chlorophyll fluorescence parameters Φ_PSII_ (photosystem II quantum yield), F_v_/F_m_ (maximum quantum efficiency of photosystem II photochemistry) and NPQ (non-photochemical quenching of chlorophyll *a* fluorescence) were calculated as Φ_PSII_ = (F_m_’-F_s_)/F_m_’, F_v_/F_m_ = (F_m_-F_o_)/F_m_ and NPQ = (F_m_/F_m_’)-1, respectively. F_o_’ was calculated according to Oxborough and Baker (1997). The coefficients of photochemical quenching (qP) and photosystem II maximum efficiency in light (F_v_’/F_m_’) were calculated as qP = (F_m_’-F_s_)/F_m_’-F_o_’ and F_v_’/F_m_’ = (F_m_’-F_o_’)/F_m_’. From CO_2_ response curves, V_cmax_ (maximum carboxylation rate), J (maximum electron transport rate at the given light intensity) and TPU (maximum triose-phosphate utilization rate) were determined after Sharkey et al. (2007). For fitting of CO_2_ response curves, mesophyll conductance was assumed to be 0.189 mol m^-2^ s^-1^, as determined for tomato (Berghuijs et al., 2015).

### Statistical Analysis

Data that had been assessed on several plants per plot were first averaged to give one response per plot. Then, averages and standard errors were calculated based on two plots per treatment (*n* = 2), and were further analyzed using analysis of variance (ANOVA). The assumptions for ANOVA, i.e., normality and homogeneity of variances, were fulfilled in all cases. As there was no systematic effect between repetitions for any of the parameters tested (data not shown), data were analyzed without taking a possible block effect into account. Motivated by the small number of experimental units (*n* = 2), treatment effects were tested at the 10% probability level as is normal in such cases (Ott and Longnecker, 2010). In the ANOVA, it was additionally tested whether a polynomial model could explain the effect of the percentage of blue supplemental light (B) on any of the tested variables (*y*).

(3) $$y=a+b*B+C*B^{2}$$

If the test was significant for parameter *c*, then there was a quadratic (i.e., nonlinear) effect of %B on the variable. If the test was only significant for parameter *b*, then %B had a linear effect on the variable. Genstat (18th edition, VSN International LTD, Hemel Hempstead, United Kingdom) was used for all statistical tests.

## Results

### Realized Daily Light Integrals and Percentages of Blue Light

**FIGURE 2:**
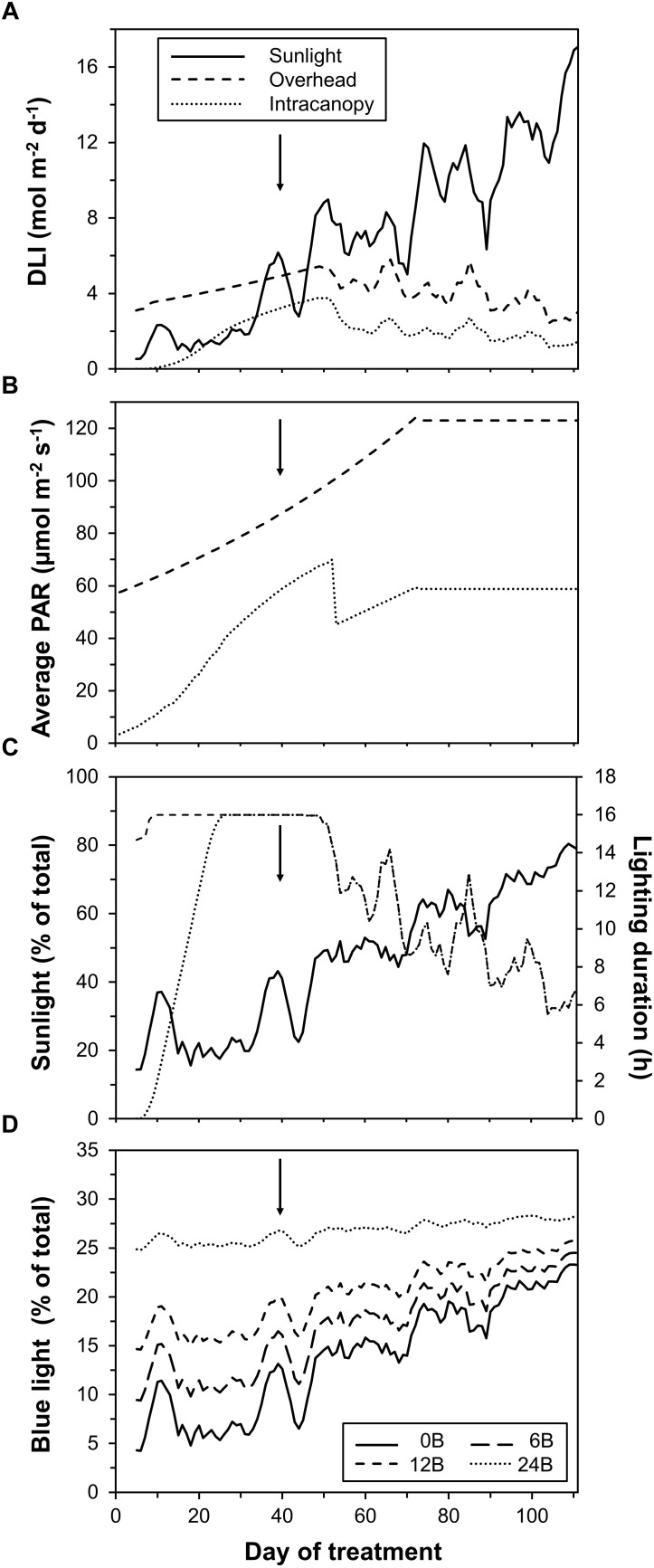
Characteristics of light incident on the crop. **(A)** daily light integral (DLI) from sunlight, overhead lighting, and intracanopy lighting; **(B)** average photosynthetically active radiation (PAR) from overhead and intracanopy lighting; **(C)** percentage of solar light and hours of lamp use; and **(D)** realized percentage of blue light (all light sources). A moving average filter across five data points was used for better visibility except for data shown in **B**. Arrows indicate time of intermediate harvest.

Until the intermediate harvest (40 DOT), plants mostly received light emitted by LEDs (∼70% of total DLI; Figure 2A). Light incident on plants and emitted by overhead LEDs increased strongly until ∼70 DOT due to plants growing toward these lamps, while light from intracanopy LEDs increased initially and decreased on DOT 53 as lamps were shifted upward (Figure 2B). The realized percentage of blue light (as percentage of total light) was 8, 12, 17 and 26% in the 0B, 6B, 12B, and 24B treatments in the initial 40 days, respectively. Thereafter, sunlight as total fraction of DLI increased (Figures 2A,C). This was due to (i) intensity and day length of sunlight increasing as a function of time of year and (ii) lamps being progressively used less throughout the day as lamps were switched off when solar intensity incident onto the greenhouse exceeded a threshold (Figure 2C). On average, over the complete experiment, sunlight contributed 54%, overhead lighting contributed 31.5% and intracanopy lighting contributed 14.5% of total DLI. The fraction of blue light increased over time due to increases in the contribution of sunlight to total DLI (Figure 2D). This increase was stronger when the percentage of blue in LED light was lower (Figure 2D). On average, the realized percentage of blue light was 14, 17, 20, and 27% in the 0B, 6B, 12B, and 24B treatments, respectively.

### Crop Growth and Development

**FIGURE 3:**
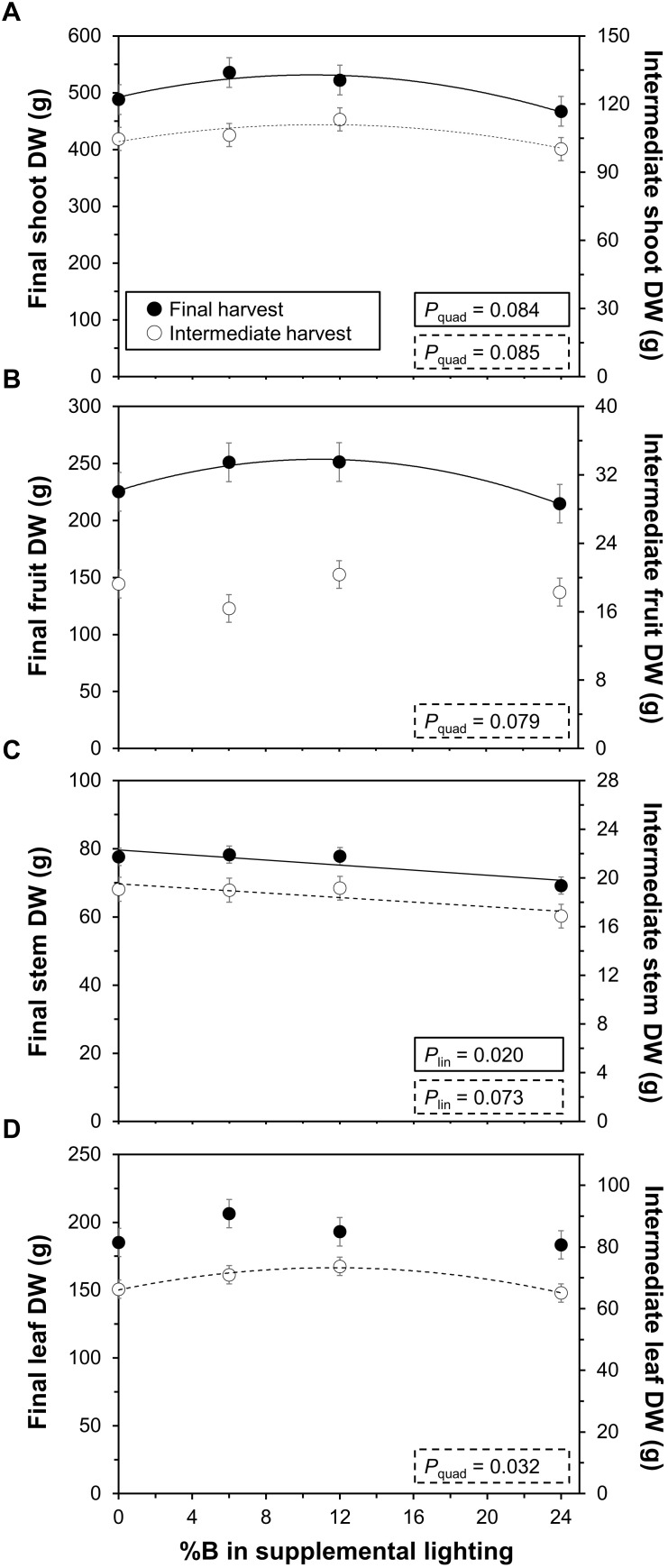
Effects of percentage of blue light in supplemental lighting on biomass per plant. **(A)** total shoot dry weight (DW); **(B)** fruit DW; **(C)** stem DW; and **(D)** leaf DW. Final harvest occurred 111 DOT, intermediate harvest 40 DOT. Data include periodically picked ripe fruits and old leaves. For significant quadratic or linear effects of supplemental blue light, a trendline together with the respective *P*-value is depicted. Data gathered from three plants per plot were averaged for one value per plot. The treatment average ± SEM was then calculated based on values from two plots per treatment (*n* = 2).

Total shoot biomass (i.e., dry mass) displayed a significant quadratic response to %B at final (*P* = 0.084) and intermediate harvests (*P* = 0.085; Figure 3A), indicating that adding blue to monochromatic supplemental red light increases biomass up to an optimum. This trend was similar for fruit DW at the final (*P* = 0.079), but not the intermediate harvest (*P* = 0.24; Figure 3B). Biomass of two out of three trusses harvested on DOT 81, 91, and 99 also showed an optimum response with %B (Supplementary Figure S1), similar to the trend seen in Figure 3B for the final harvest. Stem DW at both the final (*P* = 0.02) and intermediate harvest (*P* = 0.073) showed significant linear decreases with %B (Figure 3C), which equalled an effect size (% change between 0B and 24B treatments) of 11% at both harvests. Leaf DW showed a significant optimum (quadratic) response at the intermediate (*P* = 0.032), but not at the final harvest (*P* = 0.26; Figure 3D).

**FIGURE 4:**
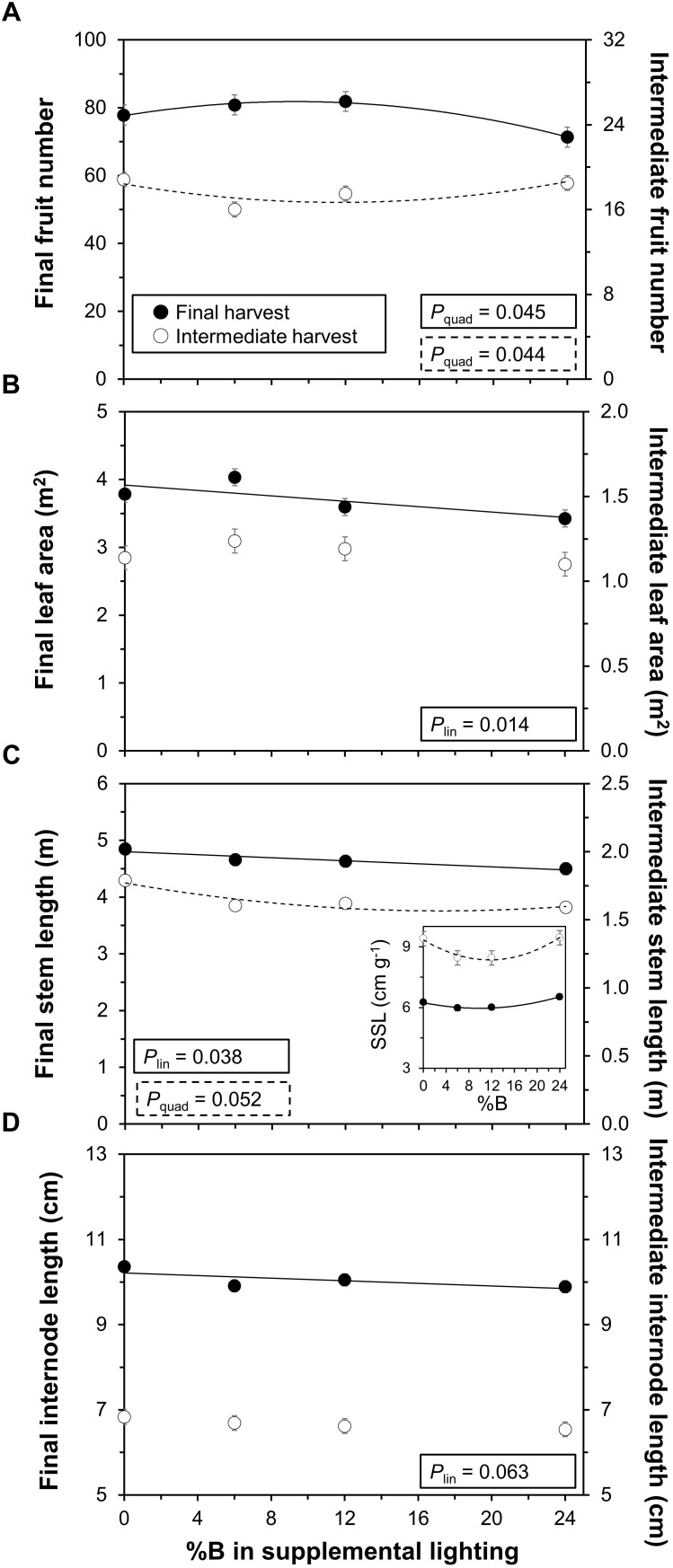
Effects of percentage of blue light in supplemental lighting on crop characteristics per plant. **(A)** number of fruits; **(B)** leaf area (LA); **(C)** stem length; and **(D)** internode length. Final harvest occurred 111 DOT, intermediate harvest 40 DOT. Number of fruits includes periodically picked ripe fruits. Inset in **(C)**: Specific stem length (SSL; stem length/stem dry weight) at final (*P*_quad_ = 0.013) and intermediate harvest (*P*_quad_ = 0.024). For significant quadratic or linear effects of supplemental blue light, a trendline together with the respective *P*-value is depicted. Data gathered from three plants per plot were averaged for one value per plot. The treatment average ± SEM was then calculated based on values from two plots per treatment (*n* = 2).

The number of fruits showed an optimum response to %B at the final harvest (*P* = 0.045), while the opposite trend (i.e., quadratic response with minimum at intermediate %B) was visible at the intermediate harvest (*P* = 0.044; Figure 4A). Leaf area decreased linearly with %B at the final harvest (*P* = 0.014; effect size: 12.2%), while plants at the intermediate harvest showed no significant response (*P* = 0.35; Figure 4B). Stem length decreased linearly with %B at the final harvest (*P* = 0.038; effect size: 6.6%), while at the intermediate harvest there was a significant downward curvature (*P* = 0.052; Figure 4C). Stem length per unit stem weight, specific stem length (SSL), showed a significant downward curvature at both the final (*P* = 0.013) and the intermediate harvest (*P* = 0.024), and thus tended to be smallest under intermediate %B treatments (Figure 4C, inset). Internode length showed a significant linear decrease at the final (*P* = 0.063; effect size: 3.6%), but not the intermediate (*P* = 0.47), harvest (Figure 4D). While time to flowering or time to fruit set were not significantly affected by the treatments, fruits initially ripened slightly faster in 0B and 24B compared to intermediate blue light treatments (data not shown), but this did not confer higher overall yield (Figure 3B). Biomass partitioning among above ground organs (based on dry weights of leaves, stems and fruits), number of leaves and specific leaf area were not significantly affected by %B (data not shown).

### Photosynthetic Gas Exchange and Chlorophyll Fluorescence

**FIGURE 5:**
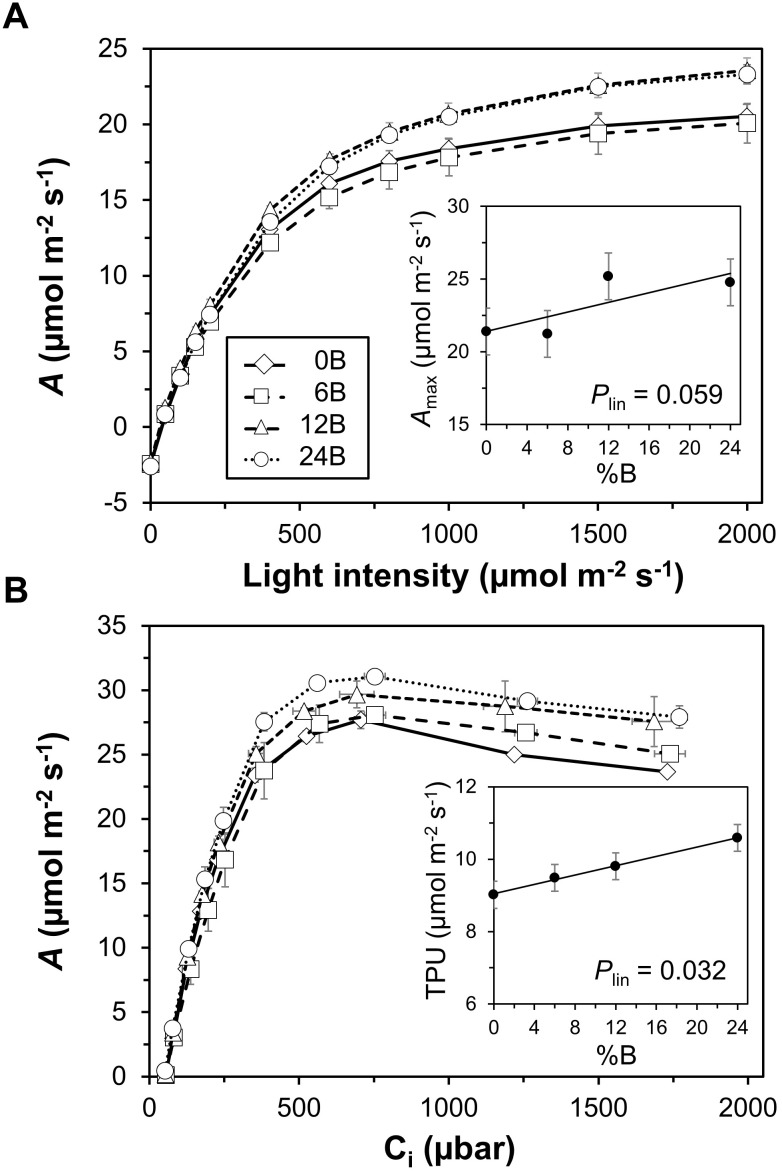
Response curves of net photosynthesis rate (*A*) to light intensity **(A)** and leaf internal CO_2_ partial pressure (C_i_; **B**). Insets: **(A)**, relationship between light-saturated net photosynthesis rate (*A*_max_) and percentage of blue light used in supplemental lighting (%B); **(B)** relationship between maximum rate of triose phosphate utilization (TPU) and %B. Trendlines together with *P*-values depict significant linear effects of %B on *A*_max_ and TPU. Data were recorded 25–27 DOT. Data gathered from three plants per plot were averaged for one value per plot. The treatment average ± SEM was then calculated based on values from two plots per treatment (*n* = 2).

Net photosynthesis rate (*A*) in leaves grown in different treatments showed very similar responses to light intensities below 200 μmol m^-2^ s^-1^ (Figure 5A). At higher light intensities, the 12B and 24B treatments tended to display higher *A* than the 0B and 6B treatments (Figure 5A). Below ∼500 μbar CO_2_, the relationship between *A* and leaf internal CO_2_ partial pressure (C_i_) was similar between treatments, but at higher C_i_, *A* tended to increase with %B. Thus, *A* tended to be lowest in leaves grown under low %B for a given value of C_i_ (Figure 5B). Increasing %B significantly and linearly increased leaf photosynthetic capacity: both *A*_max_ (*P* = 0.059; effect size: 18.6%) and TPU (*P* = 0.032; effect size: 17.1%) scaled linearly with %B (Figures 5A,B, insets). Likewise, F_v_/F_m_ showed a linear increase with increases in %B (Supplementary Figure S2A, inset; *P* = 0.094; effect size: 5.5%). Photosystem II quantum yield (Φ_PSII_), the coefficient of photochemical quenching (qP) and photosystem II maximum efficiency (F_v_’/F_m_’) reflected the increase in photosynthetic capacity with increases in %B: when measured at high light intensity, these parameters were largest in leaves grown under high %B (Supplementary Figure S2). Non-photochemical quenching (NPQ), on the other hand, was less clearly affected by %B (Supplementary Figure S2B). Day respiration (R_d_) showed a significant downward quadratic response (*P* = 0.076) with increases in %B, i.e., R_d_ was highest in 0B and 24B treatments (Supplementary Figure S3). Other photosynthetic parameters determined from light (Φ_CO2_ and Θ) and CO_2_ response curves (V_cmax_, J) were not significantly affected by %B (data not shown). Also, treatments did not affect the relationship between instantaneous net photosynthesis rates and the prevailing light intensities of the different treatment spectra (Supplementary Figure S4).

## Discussion

Different percentages of blue in the spectrum of supplemental light (%B) had significant effects on the biomass of greenhouse-grown tomato, as well as on leaf photosynthetic and chlorophyll fluorescence characteristics. The implications of our findings are discussed below.

### Lack of Blue Supplemental Light in a Greenhouse Does Not Trigger the “Red Light Syndrome,” While Increasing Blue Supplemental Light Produces Sun-Type Leaves

The “red light syndrome” is evoked when plants are grown at 100% monochromatic red light, and symptoms of this physiological deficiency include strong decreases in photosynthetic capacity, rates of electron transport, dark-adapted F_v_/F_m_ and leaf thickness, as well as unresponsive stomata and reduced leaf pigmentation (Hogewoning et al., 2010b; Ouzounis et al., 2015; Trouwborst et al., 2016). Also, monochromatic red light has been shown to cause a lower number of chloroplasts, thinner cell walls and less spongy mesophyll tissues (Goto, 2003), resulting in leaf curling (Ouzounis et al., 2014). Leaf flattening is controlled by the phototropins phot1 and phot2, and the PKS1 and PKS2 proteins regulate leaf curling in the phot2 pathway (de Carbonnel et al., 2010; Kozuka et al., 2013). Our results show that photosynthetic capacity (*A*_max_ and TPU) and F_v_/F_m_ of leaves grown under the 0B treatment were indeed lower than those grown under higher blue light percentages (Figure 5 and Supplementary Figure S1A). However, they were certainly not low enough to classify the changes seen as part of “the red light syndrome.” Also, we did not observe leaf curling due to lack of blue supplemental light, although some leaves growing close to interlighting modules did show signs of leaf curling, but this was irrespective of treatment spectrum (data not shown). Earlier studies indicated an up to threefold decrease in photosynthetic capacity in plants grown under 100% monochromatic red light compared to plants grown under a red/blue mixture (Hogewoning et al., 2010b; Trouwborst et al., 2016), while in this study plants showed only moderate decreases in photosynthetic capacity (e.g., TPU and *A*_max_ decreased by 15 and 16%, respectively, between the 24B and 0B treatments, Figure 5). Also, leaf thickness was not significantly affected by the treatments, and no visual differences in leaf pigmentation were observed (data not shown). Notably, photosynthesis and chlorophyll fluorescence were measured rather early in the experiment (25–27 DOT), when sunlight contributed less to the total DLI than later on in the experiment. Consequently, the realized spectrum in the 0B treatment contained 8% blue light in this initial period (Figure 2C), which was clearly sufficient to prevent the “red light syndrome” from occurring.

It is well established that addition of blue to red light promotes leaf expansion, reverses morphological abnormalities and promotes stomatal opening and therefore access to CO_2_, ultimately enhancing photosynthesis (Goto, 2003; Boccalandro et al., 2012; Savvides et al., 2012). As expected, the photosynthetic capacity, the efficiency of electron transport, and dark-adapted F_v_/F_m_ increased with increases in %B (Figure 5 and Supplementary Figure S1A). These results are in agreement with those of Hogewoning et al. (2010a; 2010b), which show that photosynthetic capacity as well as Φ_PSII_ show strong linear increases with increases in blue light (up to 50% blue light), producing “sun-type” leaves even in low light intensities. These increases are likely caused by larger amounts of Rubisco, cytochrome b_6_f complex, chlorophylls, and photosystem II proteins (Matsuda et al., 2004).

### Too Little and Too Much Blue Supplemental Light in Greenhouses Is Suboptimal for Growth and Yield

The ratio of red to blue light is an important factor in commercial LED applications, as both installation and use of blue additional to red light cause extra costs. Compared to monochromatic red or blue light, mixing red and blue light has generally been shown to increase yield and biomass in experiments without solar light (Goins et al., 1997; Goto, 2003; Massa et al., 2008), but less is known about the effects of partially replacing red with blue light in several blue intensities in a broad-spectrum background (Hernández and Kubota, 2014). Thus, our results add to previous research that biomass and yield respond with an optimum to %B in tomato (Figure 3). Based on our data, it seems that lower biomass under low blue light intensities is caused by different factors than lower biomass under high blue light (discussed below). However, it should be stressed that the current results were obtained from one experiment under a specific set of environmental conditions, so repetitions should be carried out before any definitive conclusions can be drawn.

Lower biomass in the 0B treatment (relative to 6B and 12B) occurred despite the plants being the tallest in this treatment (Figure 4C), and despite a comparably large leaf area (Figure 4B). Both phenomena (long stems, large leaf area) may thus have increased crop light interception (Sarlikioti et al., 2011), but this clearly did not increase biomass. This points to the possibility of lower light use efficiency under low blue light, and it is indeed striking that leaf photosynthetic capacity (i.e., TPU, *A*_max_) of leaves in the top layer of the canopy was lowest in this treatment (Figure 5). This indicates that periods of high natural light intensity may have been used less efficiently to drive photosynthesis in low blue light grown leaves. Intensities of naturally occurring light can fluctuate strongly and within seconds, partially due to movement of the sun, clouds and shade created by the greenhouse construction (Li et al., 2016; Kaiser et al., 2018), and these transient phases of high light intensity may have been used less well in the 0B treatment. Also, leaves in the 0B treatment had relatively high rates of day respiration (Supplementary Figure S3), which may additionally have lowered diurnal carbon gain. While dark-adapted F_v_/F_m_ was comparably low (0.74; Supplementary Figure S2A), indicating relatively higher photoinhibition in these leaves, this did not decrease Φ_CO2_ under steady-state light response measurements of photosynthesis, as would be expected under severe photoinhibition.

Plants under 24% supplemental blue light displayed lower total, truss and leaf biomass (Figure 3) in conjunction with smaller leaf area and shorter stems (Figures 4B,C). Consequently, it is likely that in the 24B treatment less light was intercepted by the crop. Another factor that could have negatively impacted growth is a decrease in photosynthetic light use efficiency, as Φ_CO2_ of blue is lower than that of red light (McCree, 1972). When measuring Φ_CO2_ under light-limited conditions and at the treatment percentages of blue light, however, we did not observe a difference between treatments (*P* = 0.133; Figure 5A). Also, the relationship between instantaneous *A* and incident light intensities was not affected by %B (Supplementary Figure S4). Notably, Hogewoning et al. (2010b) did not observe differences in Φ_CO2_ in leaves grown and measured under 7–30% blue supplemental light, either. It therefore seems that here, differences in light interception had larger impacts on growth in the 24B treatment than differences in leaf area-based light use efficiency. The increase in photosynthetic capacity in leaves grown under high blue light intensities (Figure 5), which would have increased *A* at high light intensities, clearly did not outweigh the likely reduction in light interception in the 24B treatment. Also, R_d_ was comparably high in leaves grown at 24% blue light (Supplementary Figure S3), which may have additionally decreased carbon gain.

### Implications for Practice and Research

The experiment described here was conducted from the middle of February until the end of May in an experimental greenhouse in the Netherlands. Because this specific experimental setup had a large number of overhead LED modules, light transmissivity of the greenhouse was relatively low (26%), which strongly decreased natural DLI inside the greenhouse and therefore conditions in the greenhouse were more comparable to Dutch winter conditions in a modern production greenhouse with ∼70% light transmissivity. To estimate the DLI that would have occurred in a modern Dutch greenhouse in the darkest part of the year, global radiation data from the years 2011–2016 was retrieved from a weather station in Wageningen, and daily average DLI was calculated. Data for calculation of winter DLI were centered around the darkest day of the year (December 21), making the period for calculating winter DLI October 28-February 15 (111 days, same duration as the experiment). To calculate the DLI that would have occurred inside the greenhouse, winter DLI outside of the greenhouse was multiplied with 0.70, and compared with the DLI due to sunlight that actually occurred during the experiment (“DLI experiment”). The comparison showed that the two DLIs were comparable in the greenhouse (Supplementary Figure S5), especially in the first 40 days, when most measurements (photosynthesis, intermediate harvest) were conducted.

Many growth-related parameters were very similar in the 6B and 12B treatments, respectively. This includes total shoot, truss and leaf biomass, number of fruits and stem length, indicating that 6% of supplemental blue light is sufficient to achieve high productivity. This could help save energy and costs in greenhouse systems that currently use higher %B. Furthermore, future research could determine whether an even lower percentage of blue light could achieve similar results. Plants usually grow (and have evolved under) white sunlight containing, next to blue and red, 35% of “green” wavelengths (500–600 nm; Bird and Riordan, 1986). It is thus likely that plants use green light, both for photosynthesis and signaling (Smith et al., 2017). Indeed, several experiments in lettuce (Kim et al., 2004a,b; Lin et al., 2013) and tomato (Kaiser et al., submitted) suggest that partially replacing a red/blue mixture by green light can improve yield. These exciting results suggest that the optimal spectral “recipe” for protected cultivation is yet to be discovered.

## Author Contributions

LM, EH, and TO conceived and designedthe study. HG, TO, RS, and EK acquired the data. EK, HG, TO, and RS analyzed and interpreted the data. EK drafted the article. All authors revised the article critically for the important intellectual content and approved the final version to be submitted.

## Conflict of Interest Statement

The authors declare that the research was conducted in the absence of any commercial or financial relationships that could be construed as a potential conflict of interest.
