# Supplementary material for: Adding Blue to Red Supplemental Light Increases Biomass and Yield of Greenhouse-Grown Tomatoes, but Only to an Optimum
Source: Front Plant Sci. 2019 Jan 14;9:2002. doi: 10.3389/fpls.2018.02002 (PMC6339924; doi:10.3389/fpls.2018.02002)
Supplement: Supplementary file 1 [file Data_Sheet_1.docx]

**Supplementary material**

**
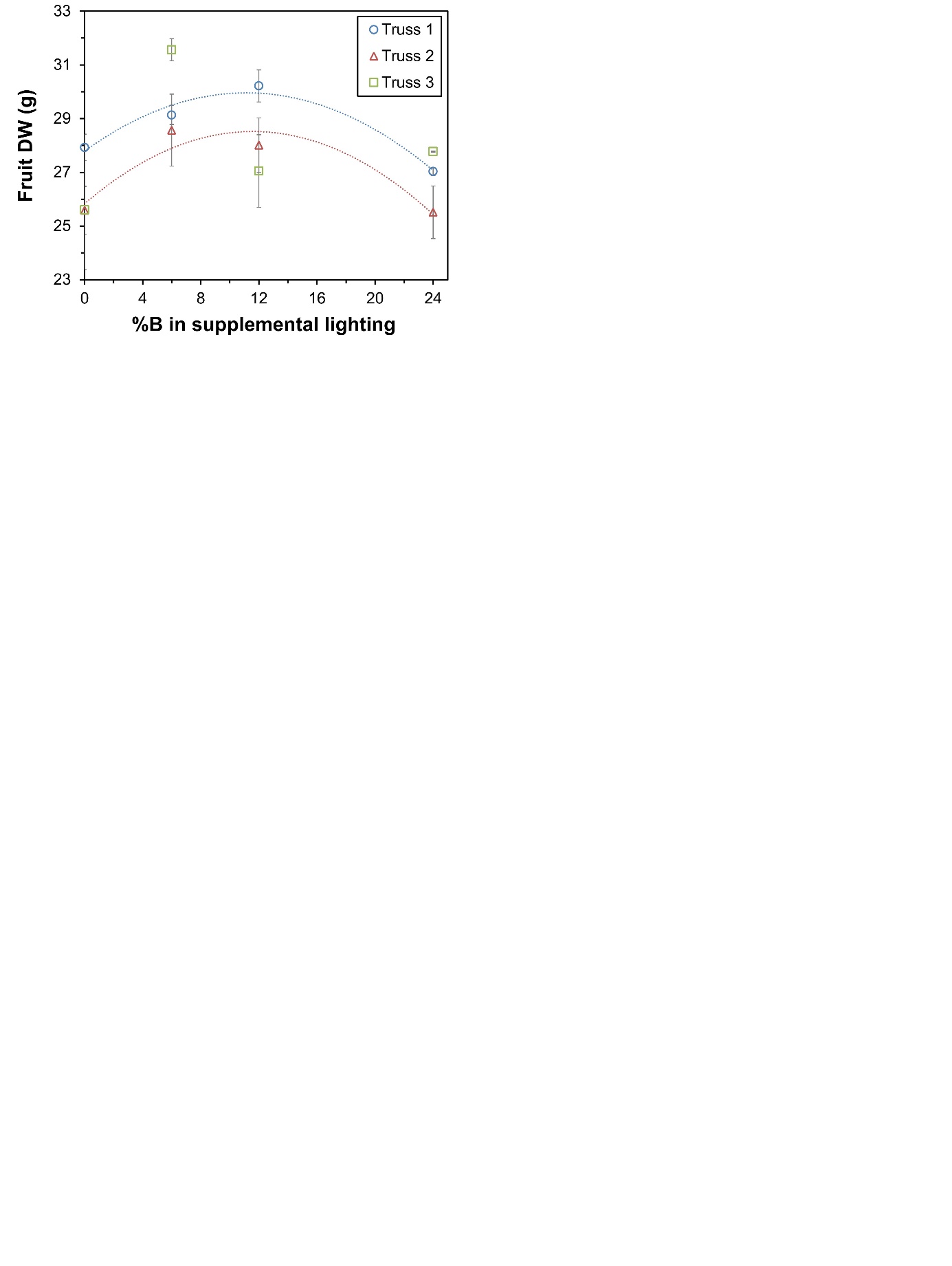
**

**Fig. S1.** Dry weight (DW) of fruits on trusses 1, 2, and 3, which were harvested on DOT 81, 91, and 99, respectively, and percentage of blue light used in supplemental lighting. For trusses 1 and 2, a polynomial relationship is shown. Data gathered from three plants per plot were averaged for one value per plot. The treatment average ± SEM was then calculated based on values from two plots per treatment (n=2).


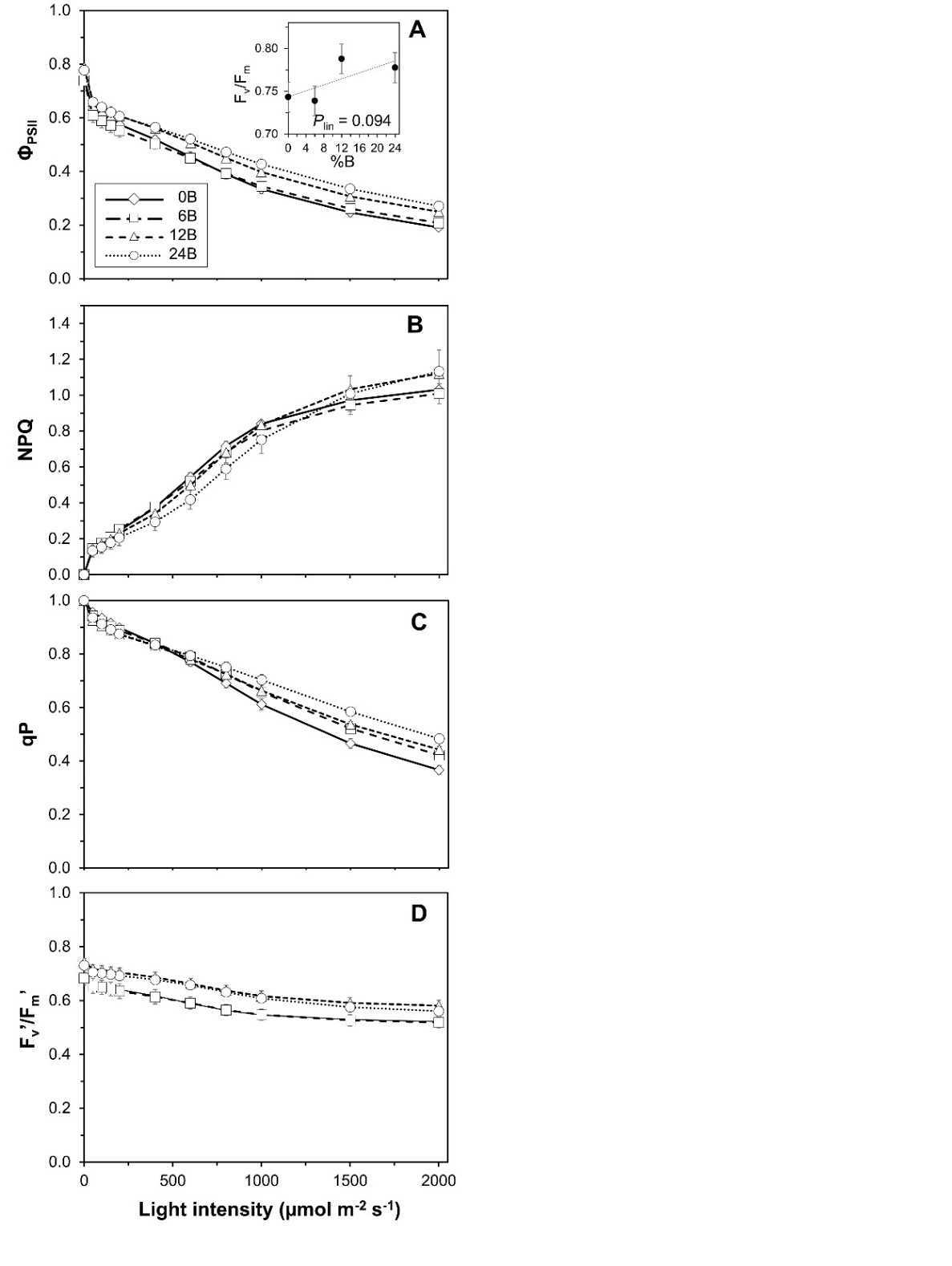


**Fig. S2.** Responses of chlorophyll fluorescence to light intensity. A, photosystem II quantum yield (Φ_PSII_); B, non-photochemical quenching of chlorophyll *a* fluorescence (NPQ); C, coefficient of photochemical quenching (qP); D, photosystem II maximum efficiency (F_v_^’^/F_m_^’^). Inset in A: relationship between dark-adapted F_v_/F_m_ and percentage of blue light used in supplemental lighting (%B); the trendline together with the *P*-value depicts a significant linear relationship between %B and F_v_/F_m_. Data gathered from three plants per plot were averaged for one value per plot. The treatment average ± SEM was then calculated based on values from two plots per treatment (n=2).


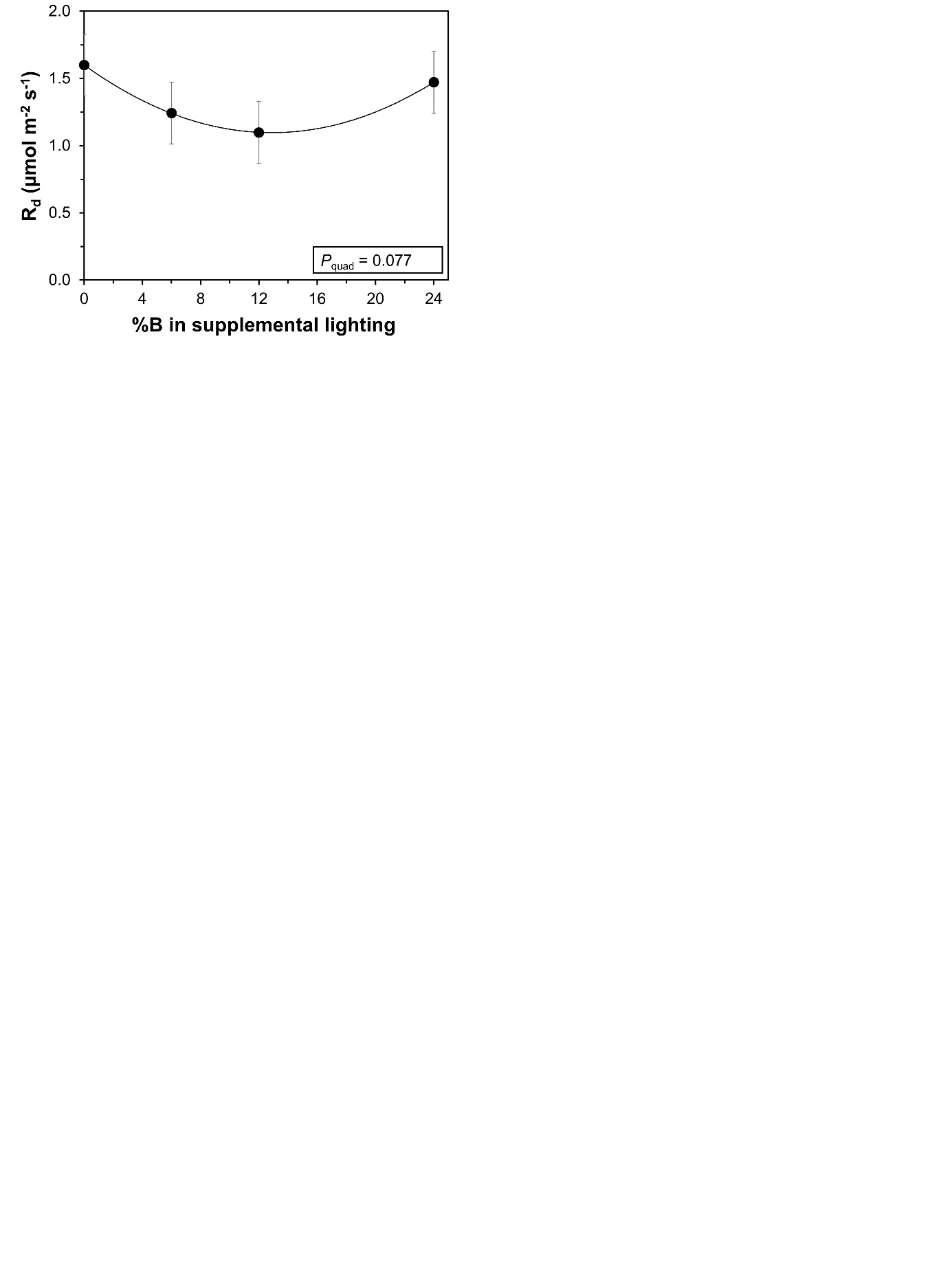


**Fig. S3.** Relationship between day respiration (R_d_) and percentage of blue light used in supplemental lighting. The trendline together with the *P*-value indicates a significant quadratic relationship between %B and R_d_. Data gathered from three plants per plot were averaged for one value per plot. The treatment average ± SEM was then calculated based on values from two plots per treatment (n=2).

**
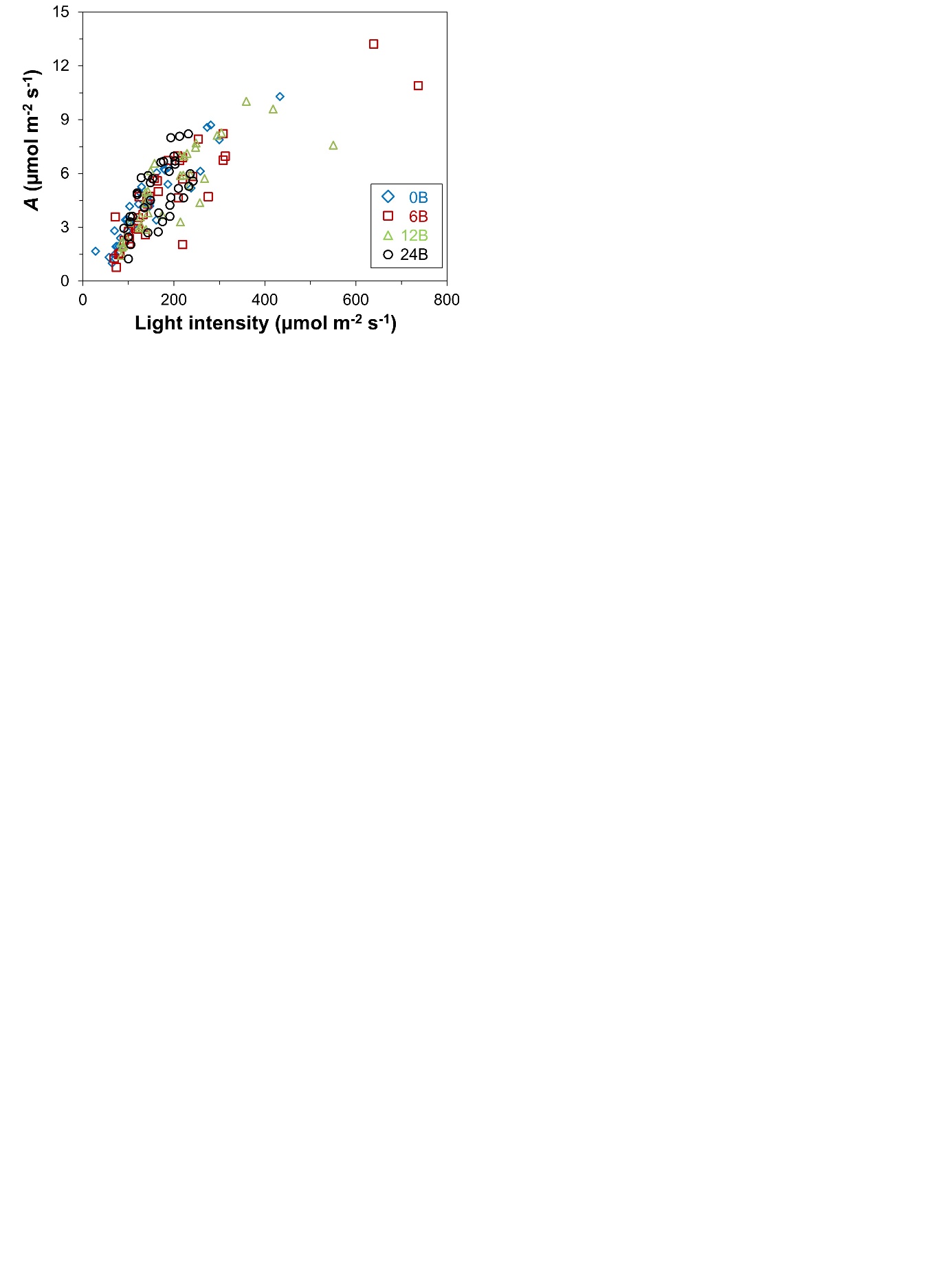
**

**Fig. S4.** Instantaneous responses of net assimilation rate (*A*) to light intensity in leaves grown under several %B treatments. Measurements were conducted 32-33 DOT on young, fully developed leaves of three plants in both plots and repeated six times (between 08:00 and 18:00 hrs). Data points represent single measurements.


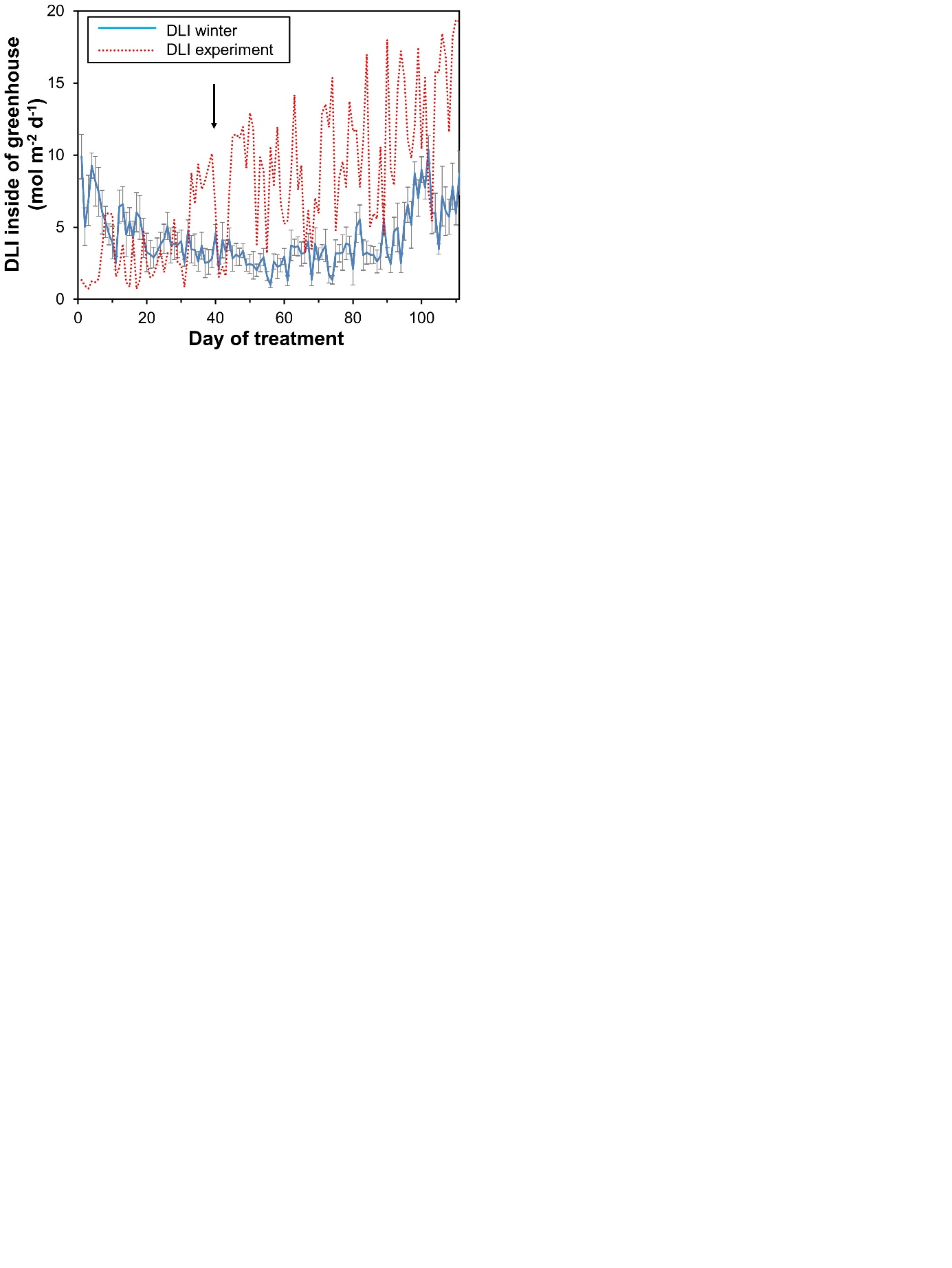


**Fig. S5.** Daily light integral (DLI) due to sunlight inside the greenhouse during winter months, assuming 70% greenhouse transmissivity (DLI winter, October 28-February 15), and realized DLI due to sunlight during the experiment (DLI experiment, February 10-May 31). DLI winter is displayed as an average ±SEM of global radiation data from five seasons (2011-2016) measured at weather station ‘de Veenkampen’ in Wageningen, the Netherlands, and multiplied by 0.70 to account for light transmissivity of a modern tomato production greenhouse. Arrow indicates intermediate harvest.
